# Efficacy of Soya Protein Concentrates on the Performance and Immunity of Broiler Chickens

**DOI:** 10.3389/fvets.2020.00539

**Published:** 2020-11-05

**Authors:** Hana A. H. Zakaria, Murad R. Ata

**Affiliations:** ^1^Animal Science Department, School of Agriculture, The University of Jordan, Amman, Jordan; ^2^The National Poultry Company, Karak, Jordan

**Keywords:** soybean concentrates, broiler, performance, carcass, Newcastle disease (ND), Avian A(H5N1) influenza

## Abstract

This study examines the supplementation of soy protein concentrates (SPC) in the diet of straight run broiler chickens and its effect on their immunity and productive performance. Eight hundred Ross 308 mixed chicks were randomly assigned to four varied dietary treatments (200 birds each), four replicates per dietary treatment (50 chicks/replicate). The diets were a control without supplement (T_0_) or supplemented with T_1_ (7g), T_2_ (8g), and T_3_ (9g)/bird of 5% SPC, which replaced SBM in the basal starter diet at a rate of 5% (W/W). Each bird received a total of 300 g of the starter diet during the first 12 days of rearing and then was fed *ad-libitum* grower and finisher diets without SPC inclusion for 35 d. On day 35, 2 birds/replicate (8/treatments, 32 birds) were randomly selected and slaughtered for carcass evaluation. Results showed that 9g showed the lowest body weight (*P* < 0.05) compared to other SPC treatments. SPC addition did not improve performance. 8g had significant (*P* < 0.05) dressing %. Carcass characteristics were not affected by SPC. Newcastle disease blood titers showed significantly higher protection for 9g and 8g SPC on d 20. Avian Influenza blood titers gave the best results with (9g) at d 30, while Infectious Bursal Disease and Infectious Bronchitis blood titers were not affected by changing dietary levels with SPC. In conclusion, results indicated that supplementing soya protein concentrates to broiler chickens in the starter period has an effect on body weight and dressing %, and that it enhanced immunity against Newcastle and Avian influenza diseases.

## Introduction

Soy products, including soybean meal (SBM), are important sources of dietary protein for poultry all over the world (1). Today, a vast array of products can be derived from soybeans, and SBM is the most commonly used soybean product in livestock feed. Other products, used to varying degrees, include full-fat soybeans, soya protein concentrate (SPC), and soy protein isolate (SPI) (2). Compared with other soya bean products, the manufacture of SPC is accomplished using aqueous alcohol or enzymatic treatment protein to remove oligosaccharides, which are soluble carbohydrates from soya protein flour (1). SPC is a supplement that may be used in poultry diets to replace SBM since it contains certain benefits because of its processing. Processing increases digestibility, the absorption of nutrients, the growth rate, body weight, and weight gain^1^. SPC has decreased amounts of oligosaccharides such as (raffinose, stachyose, and verbascose). Cumulatively, these, account for approximately 6% of SBM dry matter (3). Soybean also contains higher concentrations of carbohydrates mainly comprised of oligosaccharides and non-starch polysaccharides (NSP). With monogastrics, the effect of NSP must be taken into consideration in nutrient digestion and absorption. In addition, intestinal fermentation products must be assessed for potential benefits or detriments to the host. This highlights the necessity to increase the efficiency of utilizing fibrous materials in monogastrics, which corresponds to the maintenance and improvement of animal health in antibiotic-free production systems. One method of processing, is to use ethanol/water extraction to remove oligosaccharides and part of the soluble pectins, creating a highly nutritive substance—an insoluble fraction of the NSP—which also removes the low molecular weight of carbohydrate fractions, resulting in a high-quality SPC product (4). This trial aimed to study how different levels of SPC affect the performance and immunity of broiler chickens.

## Materials and Methods

### Experimental Birds and Diets

Guidelines set forth by the Jordanian Society for the Protection of Animals include all procedures for bird handling and use in treatments. Eight hundred one-day-old, straight-run commercial broiler chick (Ross 308) were weighed on arrival and reared under standard management practices (5), in floor pens in a closed system house, where temperature, humidity, and ventilation were controlled. The poultry house was divided using a wire mesh into 16 identical floor pens (1.40 × 2.50 m) with wood shaving as litter. The trial lasted for 35 days were the starter, grower, and finisher diets were offered (Table 1A).

**Table 1A:** Ration ingredient composition of broiler diet with and without (SPC).

**Ingredients**	**Starter with (SPC)**	**Starter %**	**Grower%**	**Finisher%**
Corn	58.19	55.67	62.71	67.12
SBM (48% CP)	30.24	37.24	30.68	25.54
SPC	5.00	–	–	–
Soy oil	2.68	3.24	3.29	4.07
Limestone	1.34	1.27	1.14	1.10
MCP	1.01	1.07	0.89	0.80
DL-methionine	0.33	0.32	0.29	0.25
L-Lysine	0.25	0.24	0.09	0.22
Salt	0.21	0.22	0.27	0.23
Choline chloride	0.12	0.10	0.10	0.09
Vitamins^a^	0.10	0.10	0.10	0.10
Minerals^b^	0.10	0.10	0.10	0.10
Coccidiostate	0.05	0.05	0.05	0.05
Phytase	0.02	0.02	0.02	0.02
**Analyzed nutrient content**				
Metabolizable energy Kcal/Kg	3,025	3,025	3,100	3,200
Crude protein%	22.16	22.49	19.73	17.74
Ether extract%	5.12	5.53	5.76	6.64
Ca%	1.00	1.00	0.90	0.85
Available P%	0.50	0.50	0.45	0.42
Lysine %	1.27	1.70	0.10	0.97
Methionine %	0.63	0.63	0.58	0.52

a Vitamin premix provided per kilogram of diet: vitamin A, 0.5760 vitamin K IU/Kg; vitamin B5, 19.4532 mg; tocopherol, 4.6799 mg; vitamin B_1_, 3.5016 mg; vitamin B_2_, 1.6994 mg; vitamin B_6_, 6.4911 mg; vitamin B_12_, 16 mg; Biotine, 0.1382 mg;; Folic Acid, 1.2692 mg; Pantothenic, 7.8104 mg. vitamin K3,.9071 mg;

b *Trace mineral premix provided per kilogram of diet: Iron, 62.0061mg; Zinc, 43.065 mg; Copper, 6.855 mg; Iodine,.0589 mg; Selenium, 1.3466 mg. SCP, soy bean concentrate, MCP, mono calcium phosphate*.

Phytase enzyme was added as a component of the diet since it increases protein digestibility. It breaks the phytic acid which is a phosphorus-containing acid and releases certain vital minerals such as Ca and P. The broiler chicks were randomly assigned to four dietary treatments (200 birds each), four replicates per treatment (50 chicks/replicate). SBM was partially replaced with soy protein concentrate in the basal starter diet at a rate of 5% (weight by weight) to obtain a 5% SPC diet, where each bird received a total of 300 g of the starter diet during the first 12 days, and then was fed *ad-libitum* on grower and finisher diets for 23 days without the inclusion of SPC since young birds are more sensitive to these products and results might be seen more clearly. The four dietary treatments which were offered to birds were as follows:

T0: Starter diet without SPC (Control diet).

T1: Control diet (160 g/bird) and starter diet with 7g SPC (140 g/bird)

T2: Control diet (140 g/bird) and starter diet with 8g SPC (160 g/bird)

T3: Control diet (120 g/bird) and starter diet with 9g SPC (180 g/bird).

SPC composition is shown in Table 1B. A standard vaccination program was applied during the experiment. Infectious Bursal Disease (IBD) vaccine was administered in ovo, while Avian Influenza (AI) and Newcastle Disease (ND) were administered at 1 day old via neck injections and a mix of spray vaccine [Infectious Bronchitis (IB) MA5 + ND clone30]. Then, on day 15, another spray vaccine was given (IB 4/91+ND clone 30). Each experimental diet was formulated according to the nutrient specifications of the Ross 308 (5) and (6), and diets were analyzed accordingly (7). Light was offered (23L: 1D) daily and feed and water were provided *ad-libitum* throughout the experiment. Birds were weighed on arrival and every week thereafter until the end of the trial.

**Table 1B:** Composition of (SPC) (soybean concentrate) on as fed basis.

**Chemical composition %**	
Protein	56.00
Carbohydrates	23.20
Water	8.00
Ash	6.80
Crude Fiber	3.50
Fat	2.50

*Hamlet Protein Company, Denmark*.

### Growth Performance, Carcass Evaluation, and Blood Sampling

Each week, feed intake, body weight, weight gain, and feed conversion ratio were measured. The difference in weight between the amount of feed offered and the feed left in each pen at the end of the week was the calculation method used to record feed intake. The difference in body weight between the end and the beginning of the week was the method used to record weight gain. By dividing feed intake by weight gain, we calculated the feed conversion ratio, then results were corrected for mortality.

At the end of the trial, two birds from each replicate (8/treatment, 32 birds) closest to the group mean body weight were randomly selected, weighed, and slaughtered after 12 h of feed deprivation in accordance with the Animal Welfare Regulations in Jordan. Next, the broilers were scalded in a 55°-60°C water tank for 30s; feathers were mechanically plucked in a rotary drum picker and eviscerated. To obtain post-slaughter hot carcass weight, the liver, heart, gizzard, feet, and head were removed, and the remaining carcass was immediately weighed. The liver, heart, and gizzard, in addition to the fat pad relative to body weight, were weighed. After that carcasses were tagged and chilled in ice-water for 1 h, they were individually packed in polyethylene bags and kept in the refrigerator for 12 h. They were thereafter weighed to obtain the cold carcass weight. The commercial parts yield was obtained by cutting parts off the carcass (neck, back, wings, legs, and breast). The cuts were expressed as a percentage of final (live) body weight.

At age 10, 20, and 30 days old, blood samples were collected from the wing vein and titers for Newcastle Disease (ND), Infectious Bronchitis (IB), and Avian Influenza (AI) infections were measured by using a Hemaglutination inhibition test (HAHI). Blood titers for Infectious Bursal Disease (IBD) on day 0 and the same ages mentioned above was measured using Synbiotics kits-USA, ELISA Reader-Bioteck-ELx800 by the Enzyme linked immunoassay test (ELISA) procedure.

### Sampling and Analysis

#### Diets

Diet samples were ground by POLYMIX grinder (model PX-MFC 90 D), (KINEMATICA AG, Switzerland) and put through a 1 mm screen. Thereafter, the samples were put in a forced air oven at 105°C for 6 h and analyzed for dry matter. The samples were then tested for proximate components and classified (crude protein, crude fiber, ether extract, and ash contents) by chemical analysis (6). Crude protein was detected following the Kjeldal method of nitrogen determination. Ash content was calculated by complete combustion (for 1 h at 200°C, then for 6 h at 600°C) using a muffle furnace (Nabe, Germany). The ether extract was determined with petroleum ether using the Soxhelt method (Gerhardt, Germany), while crude fiber was determined by (ANKOM 220 FIBER ANALYZER) by ANKOM TECHNOLOGY, USA.

#### Statistical Analysis

All statistical analyses were obtained using the Statistical Analysis System (8) (Statistical Analysis System User's Guide: Statistics version 9.1. Cary, NC: SAS Institute) as a completely randomized design (CRD). Standard means were separated by Duncan's mean separation. All variables were evaluated by pen means as the experimental unit. The statistical significance was set at *P* < 0.05 overall. The model included an analysis of the effect of the treatment on the main variables. The data were tested for the main effects of dietary treatments.

## Results and Discussion

### The Effect of Soya Protein Concentrates (SPC) on Performance

The overall growth of the broiler chicks fed with an SPC-supplemented diet showed no significant improvement. This is a surprising finding since young chicks are known to be very sensitive to changes in their diet. Table 2A revealed that feed intake was not affected by SPC partial replacement. Results agreed with those cited by (9), who demonstrated that feed intake of chicks that were fed maize-with different levels of a SPC basal diet were not affected. However, these results disagreed with those cited in a study (10) which found that there was a linear increase in feed intake in chicks fed different levels of SPC produced by fermentation, which replaced a basel maize SBM diet, with SBM levels at 50, 100, or 150 g/kg. SBM treated with a combination of fermentation and protease hydrolysis had a greater feed intake than the untreated SBM for chicks from 7 to 28 days of age, since SPC did not contain any anti-nutritional factors, however, it did contain more digestible proteins (11).

**Table 2A:** Broilers performance.

**Parameters**	**Treatments**^****1****^ **(SPC)**				
	**T0**	**T1 (7g)**	**T2 (8g)**	**T3 (9g)**	**SEM^**2**^**
**Feed intake (g/bird)**					
*Production stages (days)*					
0–7	150.00	141.50	140.00	145.50	2.80
8–14	354.75	361.75	366.75	357.25	3.72
15–21	686.00	667.25	671.50	662.00	7.32
22–28	941.50	982.25	925.75	890.50	27.012
29–35	1120.50	1081.00	1117.75	1113.50	25.36
(0–35 days)	3202.50	3231.25	3197.00	3169.25	27.83
**Weight gain (g/bird)**					
*Production stages (days)*					
0–7	20.67	20.40	19.92	20.50	0.34
8–14	37.67	40.27	40.25	38.95	1.17
15–21	70.02	67.47	68.40	68.32	1.05
22–28	72.50	79.42	76.40	66.97	3.57
29–35	84.75	85.65	86.52	71.95	4.08
(0–35 days)	57.15	58.62	58.30	53.25	1.40
**Body weight (g/bird)**					
*Production stages (days)*					
0–7	190.00	188.75	189.50	188.75	2.29
8–14	453.75	470.25	466.50	461.25	6.21
15–21	943.00	942.50	944.75	936.00	9.69
22–28	1451.25	1498.5	1479.5	1404.5	25.57
(0–35 days)	2044.50^ab^	2098.50^a^	2085.25^a^	1908.25^b^	49.27

a, b *Means within rows with varying superscripts differ significantly (P < 0.05)*.

1 *Dietary treatments used in the trial: ^1^Quantities of starter diet with 5% of SPC: T0 (Control with 0% SPCE), T1 (7g), T2 (8g), T3 (9g)*.

2 *SEM, standard error of the mean. Level of significance was set at P < 0.05*.

In our study, no significant differences in feed intake among treatments were found. This might be because the levels of SPC used were not high enough to induce a difference between treatments in feed intake as in previous studies. Results in Table 2 also concurred that there was little difference between treatments and the control group in daily weight gain during the period of study, while (12) found that the treatment of SBM with both protease and carbohydrase had a light synergistic effect on bird weight gain compared with the separate enzyme treatments. This result gives a clear indication that the degrading activity of the proteolytic and cell wall of the enzymes used, helped to better absorb nutrients in the chicken's digestive tract, thereby resulting in better body weight gain. Another reason for the small average in daily gain can be explained by the fact that the birds did not consume enough lysine with the SPC diet during the grower period to meet their growth needs. Heating SBM decreased the oligosaccharide concentration and increased free sugars concentration, which bind to free amino acids or the amino group of lysine. This is because these sugars can bind to free amino acids or to the amino group of lysine containing the carbonyl group that forms a Maillard product that is intestinally unavailable, possibly explaining why the lysine availability was reduced and thereby decreased the growth rate (3). The non-significant results among all treatments in the daily weight gain might be referred also to the field challenge for ND at d 30 as indicated by higher blood titers that were observed later.

As for the body weight of treatments and the control through the trial, Table 2A showed that there were no differences between the 1st, 2nd, 3rd, and 4th week. While T1 (7g) and T2 (8g) treatments had the highest numerical value of body weight on the 2nd, 3rd, and 4th weeks than the control. Moreover, results in the 5th week showed significant differences among treatments in body weights *P* < 0.05in T3 (9g), which had the lowest value of body weight compared to the other two treatments of SPC. A study cited by (3) stated that the effect of the enzyme treatment of SBM on oligosaccharide disappearance and efficiency of chick growth performance increased and that diets containing (ESBM) supported an increased body weight gain over the 14 days than diets containing SBM without enzymatic treatment, because the decreased oligosaccharide concentration reduced potentially the viscosity of the digestive tract, leading to a slower passage rate (increased retention time), greater access of digestive enzymes to substrates, and more rapid diffusion of absorbable nutrients to the intestinal mucosa resulting in increased digestibility of the diet and better weight gain (3).

No significant differences were noted among treatments in the cumulative feed conversion ratio, Table 2B, in accordance with another study (13), which also reported that there were no differences among the 3 diets of (SBM) produced from high-protein (SBM-HP), low-oligosaccharide (SBM-LO), and conventional (SBM-CV) in feed efficiency.

**Table 2B:** Broilers performance.

**Parameters**	**Treatments**^****1****^ **(SPC)**				
	**T0**	**T1 (7g)**	**T2 (8g)**	**T3 (9g)**	**SEM^**2**^**
**Feed conversion ratio**					
*Production stages (days)*					
0–7	1.03^c^	0.98^d^	1.00^de^	1.00^e^	0.00
8–14	1.35	1.28	1.30	1.31	0.03
15–21	1.40	1.41	1.40	1.39	0.01
22–28	1.85	1.76	1.73	1.94	0.08
29–35	1.89	1.8	1.85	2.29	0.16
(0–35 days)	1.61	1.57	1.57	1.71	0.04
**Mortality (%)**					
*Production stages (days)*					
0–7	1.25	0.25	1.75	0.75	0.59
8–14	0.75	1.00	0.75	0.25	0.36
15–21	0.75	0.50	0.50	0.75	0.27
22–28	0.50^de^	1.00^e^	0.00^d^	0.00^d^	0.25
29–35	0.75	1.00	1.50	1.25	0.51
(0–35 days)	4.00	3.75	4.50	3.00	0.99

c, d, e *Means within rows with varying superscripts differ significantly P < 0.05*.

1 *Dietary treatments used in the trial: ^1^Quantities of starter diet with 5% of SPC (soybean concentrate): T0 (Control with 0% SPC); T1 (7g), T2 (8g), T3 (9g)*,

2 *SEM, standard error of the mean. Level of significance was set at P < 0.05*.

The weekly and cumulative mortality was not affected by changing dietary levels with SPC and had no significant difference between mortality of treatments and the control during the 1st, 2nd, 3rd, and 5th week. While, there were significant *P* < 0.05, results in mortality on the 4th week in the T1 (7g) treatment. The cumulative mortality during the whole period of rearing was 4% which is considered an indication of a healthy flock. This improvement in the digestion and assimilation of SPC and degradation of anti-nutritional factors may be beneficial to broiler health. This finding agreed with another study (14), which demonstrated a significant reduction in mortality in birds receiving diets based on soybean meal and containing α-galactosidase.

In general, the reduction of the anti-nutritional factors from the SBM by the addition of SPC to the starter diet of broilers was not a factor to improve performance.

### Carcass Characteristics

Table 3 shows that the treatments did not affect breast, legs, wings, neck, and back %. These results are congruent with findings of another study (15), which evaluated growth responses, immunity, and carcass attributes to post pellet-enzyme (KEMZYMEC/S) application to corn and SBM diets. The primary active enzyme α-galactosidase in the product tested, improved energy digestibility of SBM but had no effect on the carcass or breast meat yields. Meanwhile, the inclusion of SPC increased *P* < 0.05, dressing percentage in T2 (8g) compared to other treatments. These results are also in agreement with the findings of other studies (16–18), which reported an increased percentage in carcass yields by adding enzymes in the diet which also contributes to a higher fat deposition in the carcass. Another study (19) showed no differences in the percentage of liver, heart, gizzard, and giblets due to the different dietary fermentation methods of soybean meal on carcass traits.

**Table 3:** Dressing, cuts and giblet percentages.

**Parameters**	**Treatments**^****1****^ **(SPC)**				
	**T0**	**T1 (7g)**	**T2 (8g)**	**T3 (9g)**	**SEM^**2**^**
**Carcass data (%)**					
Dressing^3^	77.21^b^	76.90^b^	79.84^a^	77.99^b^	0.48
Breast^4^	34.38	34.08	33.82	35.04	0.50
Legs^4^	26.36	26.20	26.88	27.10	0.37
Wings^4^	10.06	9.92	9.67	10.48	0.18
Neck^4^	12.97	13.20	12.76	12.33	0.56
Back^4^	14.08	14.50	14.96	13.37	0.44
Abdominal fat^5^	1.34	1.33	1.51	1.03	0.15
Liver^6^	2.21	2.23	2.12	1.91	0.12
Heart^6^	0.050	0.48	0.50	0.43	0.03
Gizzard^6^	1.43	1.60	1.34	1.30	0.13
Giblets^6^	4.15	4.41	3.96	3.65	0.26

a, b Means within rows with varying superscripts differ significantly (P < 0.05)

1 *Dietary treatments used in the trial: ^1^Quantities of starter diet with 5% of SPC (soy bean concentrate): T0 Control with 0% SPC: T1 (7g), T2 (8g), T3 (9)g*.

2 *SEM, standard error of the mean. ^3^Level of significance was set at P < 0.05*.

3 *Dressing %= cold carcass wt. /live weight×100%*,

4 *cuts % = cut wt./cold carcass wt×100%*,

5 *Abdominal fat% = Abdominal fat wt./live weight×100%*,

6 *Giblets% = Giblet wt. (g)/live wt.×100%*.

## Soya Protein Concentrates (SPC) and Immunity

### Newcastle Disease (ND)

Different SPC levels lead to significant changes in antibody titers at day 10 of age against Newcastle virus vaccine for broilers with a significant high level for T0, T1 (7g), and T2 (8g), compared to T3 (9g), *P* < 0.05 as shown in Table 4. Alternatively, the significant *P* < 0.05 high level on day 20 of age against the Newcastle virus vaccine was for T3 (9g). However, these differences disappeared among treatments on day 30 of age with a field challenge for the ND virus. The decline in the antibody titers values from days 0 to 10 of age was normal because it returned to maternal immunity, while those different values among treatments on day 20 of age were due to the effect of live vaccine with the high level of SPC addition. Another study (20) reported that there was an increased serum level of IgM and IgA, owing to the addition of fermented SBM, leaving IgG content unaffected. However, more pronounced immune responses were present in cases using a high level of SPC, indicating there is an added benefit from SPC rich in small-sized peptides, and organic acids.

**Table 4:** Titers of diseases.

**Parameters**	**Treatments**^****1****^ **(SPC)**				
	**T0**	**T1 (7g)**	**T2 (8g)**	**T3 (9g)**	**SEM^**2**^**
**Newcastle disease**					
*Production stages (day)*					
0	8.20	8.20	8.20	8.20	0.37
10	7.20^a^	6.80^a^	5.80^ab^	5.00^b^	0.55
20	2.80^b^	2.00^b^	3.00^ab^	4.20^a^	0.41
30	8.40	8.00	8.20	7.80	0.69
**Avian influenza**					
*Production stages (day)*					
0	9.10	9.10	9.10	9.10	0.18
10	1.60	2.00	1.00	1.80	0.83
20	0.80	0.60	1.00	0.20	0.40
30	0.80^b^	0.60^b^	0.80^b^	2.40^a^	0.47
**Infectious bronchitis**					
*Production stages (day)*					
0	5.90	5.90	5.90	5.90	0.33
10	6.80	8.20	5.20	5.80	0.80
20	6.20	6.20	6.80	6.60	0.38
30	7.60	7.20	6.60	7.00	0.37
**Infectious Bursal disease**					
*Production stages (day)*					
0	3398.00	3398.00	3398.00	3398.00	369.43
10	1864.40	2139.80	1918.40	1576.40	410.29
20	136.40	0.00	0.00	454.60	155.46
30	0.00	138.40	0.00	0.00	69.20

a, b *Means within rows with varying superscripts differ significantly (P < 0.05)*.

1 *Dietary treatments used in the trial: ^1^Quantities of starter diet with 5% of SPC (soy protein concentrate): T0 (Control with 0% SPC); T1 (7g), T2 (8g), T3 (9g)*.

2 *SEM, standard error of the mean. Level of significance was set at P < 0.05*.

Improvement in the digestion and assimilation of SPC, as well as the degradation of anti-nutritional factors, may benefit broiler health (10) as proved by the increased percentages of T cells, CD4, CD8, IgA, IgG, and IgM in the serum of broilers. However, another study (21) found that the different processing of SBM did not affect antibody titers against the Newcastle virus vaccine in the blood of broilers. The SPC has much lower anti-nutritional factors than soybean meal, particularly the number of oligosaccharides, lectins, saponins, and antigenic substances, Therefore, it is obvious in our study that after maternal immunity (0 day), the T3 (9g) and T2 (8g) had the best ND titers value at d 10 and 20 of age. Soybean contains about 8.3 mg/g lectines but SPC contains <1 ppm (0.001 mg/g) lectins. As stated in other research (22), lectins are glycoproteins that agglomerate red blood cells, while saponins are glycosides that hemolyze red blood cells. In our results, the SPC had a lower percentage of anti-nutritional factors than the SBM and this might explain the results of better immunity for ND.

### Avian Influenza (AI H9N2)

Different SPC levels showed no significant effect on antibody titers against the Avian Influenza virus vaccine for broilers at d 10 and 20 of age while results were significant *P* < 0.05on day 30 of age and the T3 (9g) was superior over the other treatments Table 4. It is well-documented that killed vaccines need around 21 days to activate the immunity against the virus (23), therefore, it is obvious that the T3 (9g) built immunity or titers for AI vaccine (killed vaccine) is faster than other treatments, due to the effect of the high level of SPC.

### Infectious Bronchitis (IB)

No effects were observed for the different SPC levels on the antibody titers against Infectious Bronchitis virus vaccine for broilers at 20 and 30 days of age while T0 and T1 (7g) tended to be higher over the other two treatments at days 10 of age (Table 4). However, this tendency was not important because it is due to maternal immunity. These results agreed with those cited by another study (21), which found that different processing of SBM did not affect antibody titers against Infectious Bronchitis virus vaccine in the blood of broilers.

### Infectious Bursal Disease (IBD)

As shown in Table 4, varied SPC levels had no significant effect on antibody titers against Infectious Bursa virus vaccines for broilers at all ages. These results agreed with those cited by other studies (21), which found that the processing of SBM had no significant effect on antibody titers against the IBD virus vaccine in the blood of broilers. Although SPC has lower anti-nutritional factors among all SBM meals, which affects negatively the immunity of broiler chickens, there were no titer values of IBD virus at all ages in this trial because the IBD vaccine was given in ovo. The titer value will usually begin after 40 days, which is after the trial described here ended. Similar results were reported by other previous studies (24, 25), which stated that multi enzyme supplementation and diets improved immune status and antibody titers of broiler chicks.

## Conclusions

No significant differences were obtained in this study for performance parameters such as BW gain, feed intake, and FCR since the level of inclusion was not high enough to demonstrate improvement. Dressing percentage was significant *P* < 0.05 for T2 (8g) among all groups.

T2 (8g) and T3 (9g) groups had a positive effect on immunity for ND blood titer on day 20 and AI blood titers on day 30 for T3 (9g). This indicates that using SPC in broiler diets led to a more pronounced higher concentration of immunity for ND and AI diseases only. However, additional studies are needed to further evaluate the effects of SPC inclusion in the grower and finisher diet with higher inclusion levels.

## Data Availability Statement

The raw data supporting the conclusions of this article will be made available by the authors, without undue reservation.

## Ethics Statement

The animal study was reviewed and approved by the University of Jordan, which recommends animal rights and welfare. This study did not cause any harm or suffering, according to guidelines set forth by the Jordanian Society for the Protection of Animals.

## Author Contributions

All persons who meet authorship criteria are listed as authors. The initials for each author are HZ and MA. HZ did the experimental design, supervised the whole work, and wrote the manuscript. MA ran the fieldwork, the data collection, the lab work, and the statistical analyses.

## Conflict of Interest

The authors and the National poultry farm declare that the research was conducted in the absence of any commercial or financial relationships that could be construed as a potential conflict of interest.

## Footnotes

^1^Available online at: http://www.Hamletprotein.dk

## Abbreviations

SPC: Soyprotein concentrates
ESBM: Enzyme treated soybean meal
ND: Newcastle Disease
IB: Infectious Bronchitis
AI: Avian Influenza
HAHI: Hemaglutination inhibition test
IBD: Infectious Bursal Disease
ELISA: Enzyme linked immunoassay test
SAS: Statistical Analysis System
CRD: Complete randomized design
NSP: non-starch polysaccharides.

## References

[1] Batal AB Parsons CM. Utilization of different soy products affected by age in chickens. Poult Sci. (2003) 82:454–62. 10.1093/ps/82.3.454 12705407

[2] Hans HS Larry LB James KD George FF Jr David CH Carl MP Nutritional Properties and Feeding Values of Soybeans and Their Co Products. Urbana: Department of Animal Sciences, University of Illinois (2008).

[3] Graham KK Kerley MS. Firman JD, Allee GL. The effect of enzyme treatment of soybean meal onoligo saccharide disappearance and chick growth performance. Poult Sci. (2002) 81:1014–9. 10.1093/ps/81.7.1014 12162338

[4] Choct M Dersjant-Li y McLeish J Peisker M Soy oligosaccharides and soluble non-starch polysaccharides: a review of digestion, nutritive and anti-nutritive effects in pigs and poultry. Asian Aust J Anim Sci. (2010) 23:1386–98 10.5713/ajas.2010.90222

[5] Ross Nutrient Specifications Broiler Guide 308. (2019). Available online at: www.aviagen.com

[6] NRC-National Research Council (NRC) Nutrient Requirements of Poultry. 9th ed. Washington, DC: National Academic Press (1994).

[7] AOAC Official Methods of Analysis. Association of Official Methods of Analysis. 17th ed. Washington, DC (2006).

[8] SAS SAS Statistically User's Guide. Version 7. Raleigh, NC: SAS; Institute of North Carolina (2000).

[9] Jiang HQ Gong M Ma YX He YH Li DF Zhai HX Effect of stachyose supplementation on growth performance, nutrient digestibility and caecal fermentation characteristics in broilers. Br Poult Sci. (2006) 47:516–22. 10.1080/00071660600827708 16905479

[10] Wang JP Liu N Song MY Qin CL Ma CS Effect of enzymolytic soybean meal on growth performance, nutrient digestibility and immune function of growing broilers. Anim Feed Sci Technol. (2011) 169:224–9. 10.1016/j.anifeedsci.2011.06.012

[11] Ghazi S Rooke JA Galbraith H Bedford MR. The potential for the improvement of the nutritive value of soya-bean meal by different proteases in broiler chicks and broiler cockerels. Br Poult Sci. (2002) 43:70–7. 10.1080/00071660120109935 12003341

[12] Marsman GJP Gruppen H Vanderpoel AFB Kwakkel RP Verstegen MWA Voragen AGJ. The effect of thermal processing and enzyme treatments of soybean meal on growth performance, ileal nutrient digestibilities, and chyme characteristics in broiler chicks. Poult Sci. (1997) 76:864–72. 10.1093/ps/76.6.864 9181620

[13] Baker KM Uterback CM Parsons Stein HH. Nutrition value of soybean meal produced from conventional, high-protein, or low-oligosaccharide varieties of soybean and fed to broiler chicks. Poult Sci. (2011) 90:390–5. 10.3382/ps.2010-00978 21248336

[14] Mathivanan R Selvaraj P Nanjappan K Feeding of fermented soybean meal on broiler performance. Poult Sci. (2006) 5:868–72. 10.3923/ijps.2006.868.872

[15] Kidd MT Morgan GW Jr Price CJ Welch PA Fontana EA α-galactosidase enzyme supplementation to corn and soybean meal diets for broilers. J Appl Poult Sci. (2001) 10:186–93. 10.1093/japr/10.2.186

[16] Wang ZR Qiao SY Lu WQ Li DF. Effects of enzyme supplementation on performance, nutrient digestibility, gastrointestinal morphology, and volatile fatty acid profiles in the hindgut of broilers fed wheat-based diets. Poult Sci. (2005) 84:875–81. 10.1093/ps/84.6.875 15971523

[17] Alam MJ Howlider MAR Pramanik MAH Haque MA Effect of exogenous enzyme in diet on broiler performance. Int J Poult Sci. (2003) 2:168–73.

[18] Leeson S Caston LJ Yunblut D Adding roxazyme to wheat diets of chickens and turkey broilers. J Poult Res. (1996) 5:167–72. 10.1093/japr/5.2.167

[19] Ari MM Ayanwale BA Adama TZ Olatunji EA Carcass traits, intestinal morphology and cooking yield of broilers fed different fermented soya bean meal based diets. Agric Sci Technol. (2012) 4:125–30.

[20] Feng J Liu X Xu ZR Wang YZ Liu JX. Effects of fermented soybean meal on digestive enzyme activities and intestinal morphology in broilers. Poult Sci. (2007) 86:1149–54. 10.1093/ps/86.6.1149 17495085

[21] Nahavandinejad M Seidavi A Asadpour L Effects of soybean meal processing method on the broiler immune system. Kafkas Universit Veteriner Fakultesi Dergisi. (2012) 18:965–72.

[22] Peisker M Manufacturing of soya protein concentrates for animal nutrition. Cah Opt Med. (2001) 54:103–7.

[23] Zarkov IS Diagnostic potential of the haem-agglutination inhibition test, the immunedifusion test and ELIZA for detection of antibodies in chickens, intravenously infected with ADuck Bulgarian05H6N2 Avian Influenza virus isolate. Bulg J Vet Med. (2007) 10:169–78.

[24] Attia YA Al-Khalaifah H Abd El-Hamid HS Al-Harthi MA El-Shafey AA. Effect of different levels of multienzymes on immune response, blood hematology and biochemistry, antioxidants status and organs histology of broiler chicks fed standard and low-density diets. Front Vet Sci. (2020) 6:510. 10.3389/fvets.2019.00510 32195272 PMC7015166

[25] Attia YA El-Tahawy WS AbdEL-Hamid E Nizza A El-Kelway Al-Harthi MA Effect of feed form, pellet diameter and enzymes supplementation on carcass characteristics, meat quality, blood plasma constituents and stress indicators of broilers. Archi Tierzucht. (2014) 30:1–14. 10.7482/0003-9438-57-030

